# International law and human security in a kaleidoscopic world

**DOI:** 10.1007/s40901-020-00109-w

**Published:** 2020-07-27

**Authors:** Alexander Gilder

**Affiliations:** Royal Holloway, University of London, Egham, UK

**Keywords:** United Nations, Human security, Humanization, Globalization, International organizations, UN peacekeeping

## Abstract

International law is being challenged by a multitude of new actors and networks that do not fit within the traditional Westphalian system. Similarly, security is increasingly undermined by, for example, economic, health, and environmental threats that can affect individuals’ daily lives and know no state boundaries. This is the kaleidoscopic world as outlined by Edith Brown Weiss. The concept of ‘human security’ has been advanced to inform decision-making on threats to security in the interest of individuals in a bottom-up manner. This article looks forward to methods that can counter what could be perceived as a legitimacy crisis in international law. First, some of the current challenges which international law faces are explained ranging from globalisation, the declining state-based order, and decentralised security threats. Second, the concept of human security is defined, and its contents expounded. Lastly, the thesis is advanced that a conceptual framework of human security can reorientate international law to be responsive to the kaleidoscopic world by using UN peace operations as an example of where human security could have a profound impact.

## Introduction

Global issues are increasingly kaleidoscopic to the extent that international law is undermined by a multitude of new actors and networks that do not fit within the traditional Westphalian system. Similarly, security is increasingly threatened by, for example, cross-border threats of violence, health, environmental crises, and more that become intertwined, can affect individuals’ daily lives, and are not halted by traditional state boundaries. For instance, terrorism in the Sahel, famine in Nigeria, the spread of viruses such as Ebola and Coronavirus, and the displacement of the Rohingya are a few examples from recent years where a broad notion of security has been undermined. International law fails to adequately respond to these challenges and implement new, bottom-up approaches where the international community is responsive to the needs identified by individuals.

This article understands the world to be “kaleidoscopic”, as argued by Edith Brown Weiss, where the actors and coalitions engaging in the international system are constantly changing.^1^ Developments are swift where crises can appear and quickly cross borders amidst an ever-connected world linked through information technology. Brown Weiss stresses that international law more than ever needs to be viewed as legitimate by both those who create it, importantly states, and those affected by it.^2^ This is because in recent years there has been unprecedented backlash against the international system, and more broadly globalisation, by states. Well known examples of this trend include the sceptical voice of President Donald Trump, the UK’s referendum to leave the European Union, and the global rise in nationalist politics. In addition, globalisation and access to information technology has resulted in the spread of fake news, unprecedented monitoring of individuals by states, more visible economic inequalities, and the contraction of space for civil society.^3^

The concept of ‘human security’ can be used as a conceptual framework to allow international law to better focus its attention on the individual and be responsive to the needs of persons affected by insecurity. The term “human security” is used throughout the article to denote the non-legal concept as espoused by both the UN Development Programme (UNDP) and Commission on Human Security which will be expounded in Sect. 3.^4^ It is argued that international actors, particularly the UN, would be able to utilise human security to advance a human-centred approach to international law and specifically, international peace and security. Human security would provide for the participation of a diverse range of non-state actors who previously have been unable or find it incredibly difficult to permeate the international system. By doing so the international system will remain relevant in this time of rapid change and pushback against the prevailing international order. The example of UN peace operations is used to demonstrate how a human security approach could positively influence the practice of the UN and their state partners toward the inclusion of individuals as participants. The approach argued for in this article does not purport to be a panacea that will enable a reformulation of international law in an iconoclastic manner.^5^ Nor does this article address the full range of discussions that can be had such as, how such a conceptual framework would be implemented, how a human security approach fits within scholarship from Third World Approaches to International Law (TWAIL) and also how human security relates to liberal peace, but there is not the space in one article to address these questions. Instead, the article aims to generate debate and future critical examination of how international law can accommodate for a rapidly interconnected and globalised world.

## The kaleidoscopic world, security and the legitimacy of international law

Individuals are not typically included in the making of international law or the decision-making of states and the normative international system has little scope for accommodating non-state actors in general.^6^ Instead the international system is viewed through the prism of geography, due to the principles of sovereignty and equality of (territorial) states, and the question is whether such a state-based, geographic approach is relevant in the twenty first century.^7^ On the one hand it is claimed that state sovereignty will remain relevant over the coming decades.^8^ However, Daniel Bethlehem has argued that,

[w]hile the geography of statehood is likely to remain at the root of the international system, it is becoming increasingly less important as people, goods, services, and funds flow across borders; as individuals and corporations engage directly with one another without the intermediation of states or of their paraphernalia; as virtual space takes on dimensions and an importance that rivals physical space in the world of transactions, communications, and other engagements; as regional and multilateral integration arrangements between states reduce the importance of boundaries; as international and non-governmental organizations proliferate and operate transnationally on the basis of technical mandates that transcend, or endeavour to transcend, narrow sovereign interests.^9^

The international system has, in practice, become progressively about attempting to regulate a diverse range of both public and private actors interacting across the globe regardless of state borders.

What is true is that international law has undergone a process of “humanization”.^10^ For instance, Ruti Teitel’s concept of “humanity’s law” provides a framework for the international community to recognise the interests of a multitude of actors, including individuals.^11^ In a similar vein, Anne Peters has identified that ‘law ultimately should be guided and justified by the concerns of the persons affected by them.’^12^ Peters argues in her expansive study that ‘an orientation towards the individual in communities justifies international law as a whole.’^13^ When examining the subjects of international law, Rosalyn Higgins rejects the notion that only states can be subjects as a legal fiction.^14^ Higgins advocates a view that individuals are participants in the international legal system but notes how individuals are ‘extremely handicapped’ with little access to the international arena.^15^ In the subsequent sections, this article will propose a framework which encourages both access to the international system by individuals and for their interests to be at the forefront of decision-making.

The author agrees with Peters that international law requires an orientation towards the individual to remain legitimate and this article sets out a possible method of achieving such an orientation for international peace and security. Consequently, the focus of this paper is on the implications of a globalised and interconnected world on individual security as opposed to the general ‘humanization’ of international law. Law in general exists to both protect and regulate values we deem worthy, one of which is security. Security is an ‘elastic and dynamic concept’ and can be understood in a variety of different ways.^16^ One very broad definition of security is that it means the absence of threats.^17^ Conventionally, the referent object of security has been the state. That is to say, if the state is able to maintain safety and order internally and is sufficiently capable of repelling external threats then those living within the state are also perceived to be secure. Since the Second World War the notion of security has been liberalised to become denationalised and both globalised and individualised.^18^ There has been increased attention given to a human-centric concept of security where the absence of threats for individuals, not only the state as a whole, has been given increased value. The rise in interventions by the UN Security Council, the creation of the international human rights law system, the codification of and enforcement of humanitarian law, UN agencies focused on global security and development, amongst other advancements have brought a postmodern concept of security to the forefront where the individual is the referent object of security, not the state. Nevertheless, a state-centric view of security remains present and states pursue their own global security goals with little regulation which may detract from the security of individuals.

Traditionally, the state was able to respond effectively to crises within its own borders and the state was the primary provider of security. The reality today is that in an increasingly globalised world an action or event in one state can either rapidly have ramifications elsewhere in the globe or a crisis that would once have been isolated can cut to the very core of a person’s ability to survive.^19^ The global value of security can thus no longer be purely regulated by states. Advocacy groups and coalitions can form on the internet nearly instantly and dissolve just as fast. Issues can trend on Twitter in an instant bringing the concerns of a previously isolated community to global attention and disappear just hours later. However, the discourse on social media can also be seized upon by persons, either locally or from overseas, seeking to undermine the credibility of a crisis. Those persons can easily and rapidly spread pernicious misinformation that threatens local communities.^20^ One potent example is the use of WhatsApp in India to spread misinformation, which has led to a number of deaths and violent attacks since 2015, but notably hitting a peak in 2018.^21^ It is increasingly difficult for states to regulate online activities and rapidly shared misinformation can quickly create a security crisis.

Climate change, for example, could be at the root of a food scarcity crisis in sub-Saharan Africa that then results in violence threatening the physical integrity of communities and massive internal displacement of persons. Such a crisis could cross-borders, create a storm of both accurate and false reports on social media, and ultimately at the centre of the situation are individuals seeking security, likely in the form of protection and assistance, from either their government or the wider international community. In a kaleidoscopic world a sub-national or national crisis can rapidly become a global security issue. For instance, where refugees flee a situation in their home country irrespective of state borders the security problem trickles over into other states and is no longer contained. The situation then becomes a topic of worldwide debate in news media and social media platforms where a variety of stakeholders are able to reach out globally to have their voices heard. Brown Weiss argues the international legal system will need to adapt to both respond to these changes and cater for new stakeholders to remain relevant and legitimate.^22^

Not only is the world increasingly kaleidoscopic but the international legal system is suffering from both international norm fatigue and backlash. An example given by James Crawford is the UK’s dispute over the finding of the UN Working Group on Arbitrary Detention in relation to Julian Assange in 2016.^23^ A few of the plethora of examples of norm fatigue and backlash include: the unclear status of Transatlantic Trade and Investment Partnership (TTIP) negotiations; US withdrawal from the Trans-Pacific Partnership (TPP); Russian unsigning of the Rome Statute of the International Criminal Court; the campaign of the African Union against the International Criminal Court which has led to high profile cases of withdrawal, and later rescinding of the withdrawals, from the Rome Statute including Burundi, The Gambia and South Africa; the UK’s withdrawal from the EU; and the US withdrawal from the Paris Agreement. The Russian seizure of Crimea is a potent example of how the tides have shifted since post-Cold War liberalism in international affairs during the 1990s towards rejection of the norms that underpin the international legal system.^24^ Coupled with backlash is dissatisfaction with international standard-setting such as scepticism surrounding the economic liberalisation agendas of the World Trade Organization (WTO) and International Financial Institutions (IFIs), and apprehension over increased Security Council activism.^25^

There has been gradual disengagement of some nations with the international system which can possibly affect the so-called sedimentary formations of international law. Crawford explains that states can remove some layers of international law’s sedimentary formation, but some are more difficult to erode.^26^ For example, top layers of multilateral treaties can be easily affected by states, for instance by choosing to withdraw, but the solid base of the sediment, the principles, norms and institutions, are more difficult to erode even amid the current political discourse. Current disengagement and backlash though does not only extend to treaties. Instead it also includes the UN, part of the sediment’s solid base. For example, US foreign policy under the Trump administration has led to funding cuts of over half a billion US dollars proudly stated as only the start by the then US Ambassador to the UN, Nikki Haley, on Twitter.^27^ The US displeasure with the UN came to head in December 2017 with attempts to coerce states in the General Assembly to vote against what they saw as an anti-US/Israel resolution declaring US recognition of Jerusalem as Israel’s capital as null and void with no legal effect.^28^ Ambassador Haley warned that she would be ‘taking names’ of states who vote in opposition to the US.^29^

Similar to the demands of the kaleidoscopic world, Crawford argues that international law will only survive if it manages to transform and develop over time.^30^ The changing attitude of states towards international law is a concurrent trend that will ultimately affect the functioning of the UN in combatting increasingly linked threats that affect the core of individual’s lives. A return “back to Westpahlia” to address the legitimacy crisis of international law has been described by Richard Collins as deeply unpopular.^31^ Instead, what is needed is ‘to ensure that international law reflects shared values that bind people together and that it provides processes that all regard as fair and as ensuring accountability by states, non-state actors, and the myriad of other actors, especially individuals.’^32^ Collins explains that the direct participation of individuals in international law-making is a chimera and that ‘the only plausible prospect appears to be to enhance the participatory role and normative influence of NGOs and other non-state actors as a nascent form of “global civil society”.’^33^ Nevertheless, participation of the kaleidoscopic world’s growing range of non-state actors, whether that be individuals or civil society groups who are more visible than ever, is crucial and this article argues the international system requires a new framework which can assist with accommodating those actors and refocus the attention of international actors toward inclusive decision-making processes. The following section will outline the content of human security which can serve as a conceptual framework for international law to be responsive to the kaleidoscopic world.

## What is human security?

The concept of human security seeks to respond to new interlinkages in peoples’ lives and can allow international law to react to the changing demands of a world where movements and the concerns of local people can be communicated across the globe in an instant. Human security has two key elements: (1) it aims to shift the referent object of security from the state to the individual giving the individual intrinsic value and placing the interests of the individual ahead of the state; (2) it gives rise to a broader view on what can cause insecurity and that many threats are interconnected and reinforcing.^34^ Past initiatives had attempted to draw attention to the linkages between peace and development and called on the international community to consider more than only traditional threats to peace. For instance, the Brandt Commission in 1980 stated ‘the basis for any world or national order must be people and respect for their essential rights’ deeming a state-centric, military focused notion of security as unfit for the world.^35^ The Palme Commission in 1982 introduced the idea of “common security” and recognised that states need to consider both economic progress to ensure the freedom from want and more traditional, military-based notions of security to ensure the freedom from fear.^36^ However, common security remained state-centric with the view that ‘all states have the right to security.’^37^ The Brundtland Commission in 1987 coined the term “sustainable development” and importantly considered the environment with analysis of available resources for the population, food security challenges, different energy sources, and the environment as a cause of conflict.^38^

The UNDP built on the work of the earlier commissions and advanced the notion of “sustainable human development”.^39^ Under a new administrator, Gus Speth, the UNDP sought to be increasingly involved in times of crisis aspiring to coordinate the UN’s peace operations, political and humanitarian efforts, and development efforts.^40^ Security became a concern for the UNDP because the needs of people during different forms of emergencies, be it a natural disaster, war, or humanitarian crisis, are inseparable from sustainable development.^41^ The result was the 1994 iteration of the UNDP’s Human Development Report which coined *human security*. The Report attempted to create a vision of human security that could be adopted by both states and the UN in furthering social development.^42^ The team behind the Report sought to create an approach that ‘focuses on building human capabilities to confront and overcome poverty, illiteracy, diseases, discrimination, restrictions on political freedom, and the threat to violent conflict.’^43^ The Report has been argued to represent a ‘broader normative shift leading to the strengthening of the position of individual human beings at the international scene.’^44^

Following the 1994 Report various middle powers including Canada, Norway, and Japan adopted human security approaches in their foreign policies.^45^ In 2001, the Commission on Human Security was created by Secretary-General Kofi Annan and published its *Human Security Now* Report two years later.^46^ The Commission defined human security as:

to protect the vital core of all human lives in ways that enhance human freedoms and human fulfilment. Human security means protecting fundamental freedoms—freedoms that are the essence of life. It means protecting people from critical (severe) and pervasive (widespread) threats and situations. It means using processes that build on people’s strengths and aspirations. It means creating political, social, environmental, economic, military and cultural systems that together give people the building blocks of survival, livelihood and dignity.^47^

The definition places a focus on pervasive and widespread threats in an attempt to narrow human security to the core threats to survival and make the concept more practical for operationalisation.^48^ Following the report, a number of human security-based initiatives were created within the UN. Namely, a Human Security Unit (HSU) and an Advisory Board for Human Security. The HSU in particular has helped promote and institutionalise the human security concept within the UN.^49^

It is suggested here that human security is based upon five principles, namely, (1) existing rights and norms, (2) a focus on the vital core identified in a bottom-up manner, (3) a concern for vulnerability and building resilience, (4) preventative protection, and (5) the empowerment of people to act on their own behalf and implement solutions to security threats.^50^ Others have argued the human security discourse requires a change in values for the international community but instead the operationalisation of human security provides a new avenue by which we can advance existing values and goals of the international system and ensure that they serve the needs of individuals.^51^

Respect for international legal norms such as human rights law and humanitarian law, the rule of law, accountability through legal mechanisms, and good governance are inherent to human security.^52^ The Commission on Human Security argues a rights-based human security approach ‘reorients humanitarian strategies towards enhancing people’s capabilities, choices and security.’^53^ The Commission articulates the concept of human security as one which allows for informed decision-making where human rights are examined in relation to a range of actors involved, not through a state-centric perspective. In that sense, human security can constructively refocus human rights responses to reflect the public interest, and not solely in relation to state obligations and state methods for realising rights.^54^ In general, international law ‘must be at the heart of human security’ and the latter encompasses disciplines of human rights law, humanitarian law, international criminal law and refugee law.^55^ Those disciplines are often seen in isolation and human security has the capacity to be a lens by which international actors examine a situation and ensure a range of legal regimes are used to protect individuals. By grounding human security in existing legal regimes, it can be utilised to give a universal, coherent framework that focuses the attention on protecting individuals from immediate and pervasive threats identified by those affected.

Human security has been criticised in the past as “nothing more than a shopping list” of wants and desires due to its wide-ranging definitions where any number of issues are securitised.^56^ The co-chair of the Commission on Human Security recognised that it would be wholly impracticable to address all possible security issues identified by a multitude of actors.^57^ To rectify this the Commission focused the attention of human security on the “vital core” which is what people hold to be the “essence of life” and “crucially important”.^58^ Consequently, the vital core is able to shift the focus on a needs basis for different individuals and groups. The Commission’s definition of human security, quoted above, speaks of ‘critical (severe) and pervasive (widespread) threats and situations’ to denote a focus on security threats which cut to the core a person’s activities and functions, and large-scale, recurrent dangers.^59^ The vital core can be varied depending on what the individuals and groups affected deem to be essence of their lives. In this way security is personalised and not rigid based on what the international community, and consequently states, determine to be the most critical and pervasive threats. If a human security approach was adopted, international actors such as the UN would need to seek views of individuals and groups to ascertain the core factors undermining security in a given crisis. The international actor concerned can then provide a response which will lead to meaningful improvements in the eyes of those affected.

The Commission further identified that particular groups can be disproportionately affected by security threats. Namely, people on the move, women, children, the elderly, the disabled, the indigenous, and the missing.^60^ When, in 2012, the UN General Assembly defined human security vulnerable people were singled out as being of particular concern.^61^ However, human security demands more than merely identifying where a group could be at exceptional risk. Martha Fineman has argued that the partial antidote to vulnerability is resilience. Resilience is defined as ‘what provides an individual with the means and ability to recover from harm or setbacks.’^62^ To embody a human security approach international actors would need to first identify the peculiarities of vulnerability in a given situation and second contribute to building resilience which would ultimately address the root cause of the vulnerability. For instance, the UN regularly empowers women to participate in politics which within a society where women have traditionally been subjugated could alleviate vulnerability in the long-term.

The final two principles, preventative protection and empowerment, are advanced to ensure people are shielded from severe and widespread threats on the one hand and on the other that people’s opportunities are enhanced and built upon to provide a future situation where individuals and communities are more resilient to threats. Protection may need to be offered to combat a range of threats, not only physical harm, and protection responses should be preventative where possible.^63^ Human security has a concern for more than threats of violence and as such protection must go further than ensuring only physical integrity. Instead an intervention could address foreseen health or environmental crises and use institution building programmes to build the capacity of the state in question to be better prepared for future risks.

The empowerment aspect of the Commission’s framework is thought to have more potential and shows how human security can respond to crises in a legitimate and focused way compared to existing security practices.^64^ Where people are adequately protected, they can be empowered to ‘make better choices, and actively prevent and mitigate the impact of insecurities.’^65^ The Commission argues that where people are empowered they can address issues locally and can mobilise others by gaining international attention for food shortages or protest human rights violations. Today over half of the world have access to the internet which gives an unprecedented opportunity for individuals to be empowered and use the internet to gain global attention for an ongoing security issue. A notable, and inspiring example is that of Alaa Salah, a student who led protests against former President Omar al-Bashir’s regime in early-2019. Photographs and videos of Salah gained worldwide attention first on social media and later in mainstream news media outlets. Salah helped mobilise a large number of people in opposition to al-Bashir’s government and the image was dubbed “the Image of the Revolution”.^66^ Powerful examples such as this demonstrate the power of social media in communicating concerns of populations to a wider audience across the globe.

## Can human security make international law more responsive to human need?

It has previously been argued that human security is a concept which is not useful for policymaking because its competing policy goals are too difficult to reconcile.^67^ One author notes it ‘has so far proven largely unworkable in practice.’^68^ However, it has conversely been claimed that the UN, with regards to peace operations, have been working on a human security-based agenda since 1999 regardless of the fact the term is absent from major policy documents.^69^ In addition, the UN has been said to have responsibility for an integrated approach to human security and ultimately ‘promote a positive concept [of peace] by addressing the underlying conditions and prerequisites for a stable and durable peace.’^70^

In a kaleidoscopic world, the legitimacy of actors such as the UN and states are increasingly challenged. The question becomes, are the current international institutions and legal system fit for purpose? As noted above, international law has developed into a system more focused on humanity and the position of individuals.^71^ Emmanuelle Jouannet, for example, argues that international law has become more than a means of social regulation, and is ‘becoming used to transform international society in order to make up for economic, social or equitable imbalances.’^72^ However, it has been predicted that over the coming decades international law will find it difficult to formally incorporate the diverse and innumerable non-state actors involved in this transformation into international institutions and legal processes.^73^ Human security would give guidance and orientation to international actors allowing them to engage with non-state actors and ultimately provide better preventative protection and the alleviation of vulnerabilities.^74^ A human security approach would seek to respond to global problems by ensuring bottom-up engagement with local actors and decentralising decision-making to open up discussions with a multitude of parties in the interests of increased legitimacy and accountability.

How human security is utilised, ‘all depends on what human security is understood to be: a political agenda for governments, a rallying cry that forges ad hoc or sustained coalitions of states on single issues, a common concern that brings together single-issue civil society groups under a uniting umbrella, an academic problem, or a new research category.’^75^ Human security is described as a concept by most literature. Therefore, human security is a collection of interrelated ideas and can be a guide to interpretation. Specifically, as an *agenda-setting* concept, it determines what are the most relevant issues and brings to the forefront neglected problems that have previously not been included in national and international security debates or have been on the periphery. Barbara von Tigerstrom has said that human security is ‘a concept that is designed to be used in a variety of ways, including in the interpretation and development of legal norms.’^76^ Furthermore, Gerd Oberleitner claims that ‘a human security approach to international law *can* reinforce and strengthen attempts to bring international law into line with the requirements of today’s world.’^77^ By using human security as an agenda-setting concept international actors will be able to react to the needs of individuals and provide effective responses that solidify the continued relevance of international law.

Following on from the arguments of von Tigerstrom and Oberleitner, Shireen Daft argues that human security can be a ‘synthesised overarching framework’.^78^ Daft says that human security can have legal character by serving as a framework for the expression of existing norms with human security providing a principled future direction for how international law tackles security threats.^79^ Daft believes this is possible if clear principles of human security are articulated with roots and relevance in existing international law.^80^ Daft’s argument lends well to the current position of states since most believe human security should be pursued under existing international legal frameworks and not through new legal obligations.^81^ Australia has already advanced the view that human security can provide a normative framework which can ensure collective actions are providing preventative protection, empowerment to build resilience, and direct benefits to populations.^82^ Other states agreed with, for example, Qatar arguing that by using a framework for human security states will be compelled to be proactive.^83^ Likewise, India stated that human security can be implemented and used as a framework to respond to current challenges, not only as a policy goal.^84^ If human security is not to be a legal concept in its own right what it can do is harness existing international law to pursue human-centric operational goals.^85^

A human-centred approach to international law is already evident in a number of developments over the last couple of decades. For instance, the 1997 Ottawa Treaty, the 1998 Rome Statute of the International Criminal Court, and the 2000 Optional Protocol to the Convention on the Rights of the Child have been argued to embody a shift toward giving greater importance to human security as opposed to national security.^86^ However, Hitoshi Nasu notes that the adoption of human-centred treaties does not necessarily mean the concept of *human security* is being implemented because states may simply address issues they consider a threat, in a manner they consider appropriate, while not taking into consideration the views of *individuals* facing insecurity.^87^

To counter this deficiency, it is proposed in this paper that international law can be focused toward the inclusion of individual views on insecurity by the UN implementing a conceptual framework of human security. Due to shared goals there is direct relevance of human security for the UN and the organisation represents an ideal vehicle for implementing a human security framework. The UN has long advanced the needs of individuals and a sophisticated international human rights framework, development programmes, and the protection of civilians by UN peacekeeping forces are evidence of this. It could be criticised that the implementation of human security is an unattainable Utopian goal but involving individuals as participants, using preventative methods of protection, and empowering people to be involved in security processes are, on some level, achievable facets of the human security concept. A human security framework would allow the UN to adapt to future security developments in the face of a new breed of crises brought by an interconnected and globalised world. In addition, the framework would provide consistency of approach by using a core set of individual-focused principles as the backbone of the organisation’s activities.

A conceptual framework of human security, in a similar manner to that argued by Daft, should be applied to the activities of the UN and importantly the resolutions of the General Assembly and Security Council. The principles of human security (i.e. its basis as a rights-based concept, the vital core, recognition of vulnerability, and preventative protection and empowerment) can have transformative potential as a conceptual framework. Human security is transformative because ‘[i]t is an idea that seeks to reopen analysis of the world’s priorities, to produce new integrated methodologies in analysing the most complex and pressing problems, and to give greater voice to individuals and communities in searching for solutions.’^88^ Human security does not entail additional legal obligations on the part of states but does represent an alternate expression of existing legal principles to create an approach where individuals are the referent object of security. Individuals may then determine which threats to security, and consequently related legal rights, require the immediate attention of the international community.

Under such a framework, the UN and its member states can open an equal dialogue with national, regional, and local actors to determine if efforts in a broad range of security-related areas are ‘having unintended negative effects on people’s security “on the ground” in spite of good intentions, or whether they are achieving the goals articulated in terms of benefit to affected populations.’^89^ Also, decision making, for instance by the Security Council, may not explicitly threaten human security but choices may be made that do not reflect the immediate needs of individuals and not respect human security. Resources may not be used most efficiently to the benefit of empowering individuals and reducing their vulnerability. It has previously been claimed that the ‘ability to link diverse issues and encourage a coordinated approach constitutes the most important contribution of a human security approach.’^90^ Drawing on Benedek’s belief that human security and human rights should not be used interchangeably but can “redefine and refine” our approaches to peace, security and human rights, a conceptual framework of human security has a role to play in the international community.

The implementation of a human security approach must be undertaken across the full spectrum of actors. At the UN-level, the UN General Assembly has adopted a definition of the concept and has also held a thematic debates on the subject.^91^ However, it is the UN Security Council which has the primary responsibility to maintain international peace and security under Article 24 of the UN Charter and can take action under Chapters VI and VII of the Charter to either settle a dispute through peaceful means or take enforcement measures to maintain or restore international peace and security.^92^ Therefore, the Security Council is unique in its position to articulate threats to international peace and security, advance what it deems to be an important agenda, and coordinate a human security approach.^93^ Trina Ng suggests that if the Security Council adopted a framework of human security then its interpretation of international peace and security would be radically altered resulting in a limitless expanded application of Chapter VII.^94^ While it is true human security would affect what the Security Council recognises as a serious threat or acute danger it would be doing so on the basis of bottom-up information from individuals and civil society. In a time where non-state actors are calling for action on various global issues it is suggested here that the Security Council should engage with a broader notion of security that is responsive to the needs of individuals even if the application of Chapter VII is altered.

States are a necessary adjunct to any security action taken by the Security Council as states are the ultimate decision makers in the procedure of the Council and troops must be contributed by states for collective security action. As such, a human security approach at the UN would necessarily need to be acceptable to states. A potential threat to this support is the above mentioned scepticism that human security should not entail additional legal obligations or erode state sovereignty. However, this is not the case as Barry Buzan reiterates that, states are a ‘necessary condition for individual security because without the state it is not clear what other agency is to act on behalf of individuals.’^95^

Human security is, in the first instance, provided by states. States must consider that they are sovereign only so long as they are effective in developing conditions and preventing conflict to provide for the well-being of individuals under their control.^96^ Human security does not disregard the importance of state infrastructure and provisions for protecting its own population. Instead, ‘strengthening the function of states is the major course of human security.’^97^ Where a state is failing and its institutions no longer functioning there can be profound damage to human security.^98^ The second level of human security is the responsibility of actors such as the UN to act in defence of human security where a state fails to do so. This second level is where the use of a human security framework by the UN Security Council fits in. The third tier of human security is where empowered individuals, communities, and civil society provide for their own security. This is where people themselves become involved in the participatory process of security.^99^ That is to say, individuals are active subjects in identifying and implementing solutions to security issues.^100^ This can be undertaken by individuals being given platforms to voice security concerns and the proposal of solutions, especially in post-conflict situations where a society is being rebuilt.^101^ The next section will provide an example of UN practice which can be informed by the concept of human security to include bottom-up engagement and an inclusive decision-making process which recognises individuals as valuable participants.

## Human security in UN peace operations

A conceptual framework of human security could have a profound effect on the mandating of peace operations by involving meaningful engagement with individuals. This is an ideal example since UN missions in the field are necessarily engaged in activities with local communities and national reconciliation efforts that would benefit from a bottom-up, human security approach to decision-making and mandate implementation. It has previously been argued by Howe, Kondock and Spijkers that while peace operation mandates do not use the language of human security they are impacted by the UN’s wider human security agenda which includes the protection of civilians, R2P and pursuit of the Millennium Development Goals.^102^ This conclusion though conflates the distinct concept of ‘human security’ with other principles. Mainly drawing on examples from the United Nations Multidimensional Integrated Stabilization Mission in Mali (MINUSMA) and the United Nations Multidimensional Integrated Stabilization Mission in the Central African Republic (MINUSCA), the beginnings of a human security approach at the UN is described with a discussion of how this could be developed further under a conceptual framework.

Section 3 outlined five principles of human security. By applying an analytical framework based upon those principles it is possible to see (1) where peace operations have sought to carry out their activities in line with human rights and the rule of law; (2) where space is provided to identify the vital core; (3) where vulnerabilities are identified and building resilience has been attempted; (4) what aspects of protection are focused on by the missions; and (5) if individuals are empowered to act on their own behalf and implement solutions to security threats.

Both MINUSMA and MINUSCA have carried out a number of activities aimed at promoting human rights and establishing the rule of law. MINUSMA has been directly involved in the training of Malian Defence and Security forces and police since 2013 and understands ‘long-term reconciliation will not be possible without the promotion and defence of the human rights of all communities in the north.’^103^ The focus on human rights, both as a task to assist the Malian authorities with and as a monitoring and reporting task for the UN, has since been carried over into each renewal of MINUSMA’s mandate.^104^ As for MINUSCA, human rights and extending the rule of law forms a crucial part of its mandate with the mission working to revitalise the justice system in the wake of widespread human rights abuses and sexual violence. A hybrid Special Criminal Court has been established to try ‘serious crimes, including serious violations of human rights and international humanitarian law, including conflict-related sexual violence as well as grave violations of the rights of the child, that constitute a threat to peace, stability or security’.^105^ Both missions have also sought to re-establish the rule of law by training judges and magistrates and reopening courts with the ultimate goal of ending impunity.^106^

Implementation of the vital core would allow the UN to determine what is crucially important for local people to be able to respond in a bottom-up manner. Missions can demonstrate a concern for the vital core by opening space for dialogue with individuals. Prior to MINUSMA’s deployment the UN’s preliminary assessment mission held talks with ‘a broad cross section of Malian society’ to make recommendations for how the UN can provide assistance.^107^ A key method of engaging with communities in both MINUSMA and MINUSCA has been through the use of community liaison assistants (CLAs). CLAs aim to improve communication with local communities and to gain ‘a better understanding of the population’s concerns and expectations of the Mission’ and are ‘critical links’ between the mission and communities in remote areas.^108^ CLAs speak the local language, can find out the risks to communities, act as mediators, and communicate outcomes to both civilian and military contingents of an operation. CLAs are able to work with communities to formulate protection strategies and alert mechanisms for community security.^109^ Through to 2017 MINUSMA remained committed to improving ‘community outreach’ in the north to respond to the needs of the local population.^110^ However, it is worth noting that while the missions contain examples of community outreach it is unclear to what extent local views on security in actuality inform the Security Council’s decisions on mandate priorities or the UN’s decisions on what initiatives it will pursue within the country as part of the mandate.

The concept of human security recognises vulnerability as a crucial factor in decision-making. The mandates of MINUSMA and MINUSCA typically recognise the vulnerability of women and children and call for specific protection and the deployment of Child Protection Advisors and Women Protection Advisors.^111^ Other groups have been identified as vulnerable in Mali and the CAR such as displaced persons,^112^ the elderly,^113^ disabled,^114^ and Muslim communities.^115^ To what extent resilience is built is another matter. Resilience can be read into some UN activities such as the creation of employment opportunities for young people, the promotion of gender equality, and the involvement of women in the peace process.^116^ However, the focus of the UN remains on protection rather than resilience building initiatives.

Both MINUSMA and MINUSCA have protection of civilian (PoC) mandates. For a human security-based analysis the question is whether the PoC mandates take preventative action and look beyond only physical harm. The PoC mandates themselves specify the protection of civilians ‘under imminent threat of physical violence’.^117^ The missions have utilised so-called ‘robust’ force to establish control of territory and deter armed groups from harming civilians.^118^ In one sense this can be seen as preventative as the UN is taking steps to mitigate harm to civilians. Another key area of protection for the missions is the prevention of sexual violence and abuse against women and children by providing training to a multitude of actors and assisting the host governments with introducing preventative measures.^119^ The missions clearly focus on physical protection but wider human security concerns can be seen where vulnerabilities identified above are linked to the type of protection offered, as with preventing sexual violence against women and children.

Empowerment requires giving agency to local people to be able to make choices for their future and demand improvements which enhances their security. The UN has made a concerted effort to empower women in Mali and the CAR to have active roles in the peace process and national politics.^120^ The Security Council acknowledges ‘the significant contribution that women can have in conflict prevention, peace building and mediation efforts.’^121^ The missions have also undertaken and supported actions that provide space for people to be empowered to act on their own behalf in identifying and implementing solutions to the crisis. For instance, local peace and reconciliation committees, initially established by MINUSCA in 2015, contain elected persons from within a community and ‘are expected to solve local conflicts and promote peace through mediation and dialogue.’^122^ This is remarkably similar to the human security aim of giving people the ability to act on their own behalf and on the behalf of others.^123^ Local peace committee activities have run community awareness campaigns, reopened markets, and facilitated dialogue between farmers and herders to resolve differences peacefully.^124^

Human security is undeniably a difficult concept to quantify. Though the examples above outline some areas of current UN practice which do operationalise aspects of human security. However, there is much more which could be achieved with the application of a conceptual framework for human security. For instance, there is an overwhelming focus of UN peace operations on protecting individuals from *physical* harm. Human security goes beyond physical protection and necessitates a concern for harms rooted in, for example, environmental, health, or economic causes. The UN Mission in Liberia (UNMIL) did provide support during an Ebola outbreak but more could have been done in this regard.^125^ The Security Council declared the Ebola outbreak in 2014 a threat to international peace and security and took a major leadership role during the crisis.^126^ That being said, UN peace operations operating in affected areas did not have their mandates modified to include the new concern for health security. Under a conceptual framework for human security, the protection of health security would have become a key concern for the UN missions in response to local needs in areas hit by the Ebola virus. Going further, if UN peace operations adopted the conceptual framework there would not need to be a global pandemic to result in UN attention on health threats. Instead, UN missions in the field would actively work on improving access to health services with other partners if that need is identified by a local population.

Value can be added to UN peace operations by orientating practice toward initiatives like informing decision-making with local views on security needs, the building of resilience to a range of vulnerabilities, and allowing for dialogue *within* communities as well as the empowerment of local people to engage on national and international levels. Under the conceptual framework, UN missions would need to explicitly identify and plan initiatives that build resilience and prioritise empowerment at the local level to ensure communities are able to resolve conflict peacefully and engage with national reconciliation. While the UN implements some wider empowerment related strategies, either on its own or in partnership with host governments, they do not feature as focal points of mission examples given above. Empowerment is fundamental for the international actor to gain a local-level understanding of the conflict and interlinkages between security threats and a conceptual framework for human security would assist the UN in achieving meaningful empowerment. This case study has served as a starting point for international lawyers to begin to consider and visualise how the concept of human security could be further implemented in other UN activities and the application of international law with individuals as participants.

## Conclusion

The concept of human security can be a valuable tool to reorientate international law, and importantly the work of the UN, toward a human-centric notion of security. By doing this, international law would remain legitimate in the face of declining state-centric attitudes and would be able to respond to new security challenges in the interlinked kaleidoscopic world. Human security would allow new actors to penetrate the international system and ensure that the existing structures remain legitimate in a time where coalitions and decentralised global movements are ever present. Particularly in situations of conflict, it is crucial that the voices of local communities, national groups and other non-state actors are able to access the global discourse in a formal manner as opposed to relying on social media traction to garner attention for their concerns. The earlier mentioned example of Sudan is one success story of how social media influenced the international community’s discussion of a crisis. However, there are countless other conflicts which to do not attract the same level of attention.

A conceptual framework of human security would provide space for the UN to engage with non-state actors in a meaningful way. The example of peace operations shows the beginnings of an approach that implicitly takes into account the principles of human security. The implementation of these principles could be expanded to ensure international responses and decision-making are locally-informed and prepare communities for future security threats. A conceptual framework of human security would provide an approach in which the UN, and states, could achieve human-centric, locally-informed peace in a globalised and interconnected world where individuals are able to permeate international discourse more than ever before. There are of course many unanswered questions and other scholars may wish to continue this debate by linking the conceptual framework to TWAIL scholarship, the women peace and security agenda, critiques of liberal peacebuilding, and much more.

## Footnotes

1 Edith Brown Weiss, ‘Establishing Norms in a Kaleidoscopic World’ (2019) 396 *Recueil des Cours de l’Académie de Droit International*; Edith Brown Weiss, ‘On Being Accountable in a Kaleidoscopic World’ (2010) 104 *American Society of International Law Proceedings* 477; Edith Brown Weiss, ‘International Law in a Kaleidoscopic World’ (2011) 1 *Asian Journal of International Law* 21.

2 Brown Weiss 2019, *supra* note 1.

3 Ibid.

4 UNDP, *Human Development Report 1994* (OUP 1994); Commission on Human Security, *Human Security Now* (New York 2003).

5 For an iconoclastic take see Matthew Nicholson, *Re-Situating Utopia* (Brill 2019).

6 Andrew Clapham, ‘The Role of the Individual in International Law’ (2010) 21 *European Journal of International Law* 25–30. For more expansive studies of the international legal personality of individuals see, Kate Parlett, *The Individual in the International Legal System* (Cambridge University Press 2011); Astrid Kjeldgaard-Pedersen, *The International Legal Personality of the Individual* (OUP 2018).

7 Daniel Bethlehem, ‘The End of Geography: The Changing Nature of the International System and the Challenge to International Law’ (2014) 25 *European Journal of International Law* 9–24, 10–1.

8 Aurel Sari and Agnieszka Jachec-Neale, ‘International Law in 2050’ (ECIL Occasional Paper 2018/1) <https://ssrn.com/abstract=3180686> 13.

9 Bethlehem, *supra* note 7, 15.

10 See e.g. Theodor Meron, *The Humanization of International* (Martinus Nijhoff 2006); Antônio Augusto Cançado Trindade, *International Law for Humankind: Towards a New Jus Gentium* (Brill 2010 1st ed).

11 Ruti Teitel, *Humanity’s Law* (OUP 2011) 216. See also, 139–165.

12 Anne Peters, *Beyond Human Rights: The Legal Status of the Individual in International Law* (CUP 2016) 553

13 Ibid. 554.

14 See Rosalyn Higgins, *Problems and Process: International Law and How We Use It* (OUP 1994) 48–55.

15 Ibid. 51.

16 Hitoshi Nasu, ‘Human Security and International Law’ In: Mary E Footer, Julia Schmidt, Nigel D White and Lydia Davies-Bright, *Security and International Law* (Hart 2016) 28.

17 Ken Booth, ‘Security and Emancipation’ (1991) 17 *Review of International Studies* 313, 319.

18 Christopher Daase, ‘“National, Societal”, and “Human Security”: On the Transformation of Political Language’ (2010) 35(4) *Historical Social Research* 22, 34. See also, Cornel Zwierlein, Rüdiger Graf, ‘The Production of Human Security in Premodern and Contemporary History’ (2010) 34(4) *Historical Social Research* 7.

19 Bethlehem has noted that there are considerable cross-border challenges related to public health, the environment and food security in light of how transboundary the world has become. See Bethlehem, *supra* note 7, 16–7.

20 Edith Brown Weiss, ‘The Emerging International System and Sustainable Development’ (2000) 1 *International Review for Environmental Strategies* 9, 11; Brown Weiss 2011, *supra* note 1, 29–30.

21 For a recent study on WhatsApp usage in India and the link to the attacks see Shakuntala Banaji and Ram Bhat, ‘WhatsApp Vigilantes: An exploration of citizen reception and circulation of WhatsApp misinformation linked to mob violence in India’ (LSE Department of Media and Communications 2019) <http://www.lse.ac.uk/media-and-communications/assets/documents/research/projects/WhatsApp-Misinformation-Report.pdf>.

22 Brown Weiss 2019, *supra* note 1.

23 James Crawford, ‘The Current Political Discourse Concerning International Law’ (2018) 81 *Modern Law Review* 1, 5–6.

24 See e.g. Phillip Remler, ‘OSCE Mediation in an Eroding International Order’ (2016) 27 *Security and Human Rights* 273.

25 Richard Collins, *The Institutional Problem in Modern International Law* (Hart 2016) 223.

26 Crawford, *supra* note 23, 21.

27 Julian Egan, ‘US funding cuts to UN agencies would be costly for peace and security’ (The Guardian, 31 Janaury 2017) <https://www.theguardian.com/global-development/2017/jan/31/un-funding-cuts-us-trump-administration>; Nikki Haley, “Just 5 months into our time here, we've cut over half a billion $$$ from the UN peacekeeping budget & we’re only getting started.” [Twitter post, @AmbNikkiHaley], 29 June 2017 <https://twitter.com/nikkihaley/status/880232833322713088>.

28 UN General Assembly, ‘Status of Jerusalem’ (19 December 2017) A/ES-10/L.22.

29 Peter Beaumont, ‘US will 'take names of those who vote to reject Jerusalem recognition’ (The Guardian, 20 December 2017) <https://www.theguardian.com/us-news/2017/dec/20/us-take-names-united-nations-vote-to-reject-jerusalem-recognition>.

30 Crawford, *supra* note 23, 22.

31 Collins, *supra* note 25, 240.

32 Brown Weiss 2011, *supra* note 1, 32.

33 Collins, *supra* note 25, 241.

34 Nasu, *supra* note 16, 29.

35 Independent Commission on International Development Issues, *North-South: A Program for Survival* (MIT Press 1980) 268; Jean-Philippe Thérien, ‘Human Security: The Making of a UN Ideology’ (2012) 26(2) *Global Society* 191–213, 197.

36 *Common Security: A Programme for Disarmament – The Report of the Independent Commission on Disarmament and Security Issues under the Chairmanship of Olof Palme* (Pan 1982) xii.

37 Hisashi Owada, ‘Human Security and International Law’ In: Ulrich Fastenrath, Rudolf Geiger, Daniel-Erasmus Khan, Andreas Paulus, Sabine von Schorlemer, and Christoph Vedder (eds), *From Bilateralism to Community Interest: Essays in Honour of Bruno Simma* (OUP 2011) 505–520, 507.

38 United Nations World Commission on Environment and Development, *Our Common Future* (OUP 1987).

39 See e.g. Craig N Murphy, *The United Nations Development Programme A Better Way?* (CUP 2006).

40 James Speth, ‘Challenges for Sustainable Human Development: Good Governance and Democratization’ (Bruno Kriesky Forum for International Dialogue, Vienna, Austria, 1997) 7.

41 Murphy, *supra* note 39, 277.

42 UNDP, *supra* note 4, 24. See also, Des Gasper, ‘Securing Humanity: Situating ‘Human Security’ as Concept and Discourse’ (2005) 6 *Journal of Human Development* 221–245.

43 Amitav Acharya, ‘Human Security’ In: John Baylis, Steve Smith and Patricia Owens (eds), *The Globalisation of World Politics* (OUP, 6th ed, 2014) 448–462, 449.

44 Veronika Bílkova, ‘Ensuring Human Security in Armed Conflicts: The Role of Non-State Actors and its Reflection in Current International Humanitarian Law’ In: Cedric Ryngaert and Math Noortmann (eds), *Human Security and International Law* (Intersensia 2014) 29–5, 30–1.

45 Tom Farer, ‘Human Security: Defining the Elephant and Imagining Its Tasks’ (2011) 1 *Asian Journal of International Law* 43, 46. See e.g. Jennifer Moher, ‘Canada’s Human Security Agenda’ (Peacebuilding and Human Security Division, Department of Foreign Affairs & International Trade, Canadian Government, December 2012) <http://odihpn.org/magazine/canada%C2%92s-human-security-agenda/>; Department of Foreign Affairs and International Trade, ‘Human Security: Safety for People in a Changing World’ (April 1999) <http://www.summit-americas.org/Canada/HumanSecurity-english.htm>; Japanese Ministry of Foreign Affairs, *Diplomatic Bluebook 1999: Japan's Diplomacy with Leadership Toward the New Century <*http://www.mofa.go.jp/policy/other/bluebook/1999/index.html> Chapter 2(3).

46 Commission on Human Security, *supra* note 4.

47 Ibid. 4.

48 Sabina Alkire, ‘A Vital Core that Must Be Treated with the Same Gravitas as Traditional Security Threats’ (2004) 35 *Security Dialogue* 359, 360.

49 Thérien, *supra* note 35, 209.

50 This framework is set out in more detail by the author in Alexander Gilder, ‘Human Security and the Stabilization Mandate of MINUSCA’ (2020) *International Peacekeeping* 1–32 10.1080/13533312.2020.1733423.

51 Matt McDonald, ‘Human Security and the Construction of Security’ (2002) 16 *Global Society* 277, 279.

52 Shireen Daft, *The Relationship Between Human Security Discourse and International Law: A Principled Approach* (Routledge 2017) 5.

53 Commission on Human Security, *supra* note 4, 27.

54 Gerd Oberleitner, ‘Porcupines in love: the intricate convergence of human rights and human security’ (2005) 6 *European Human Rights Law Review* 588, 601–2; Christian Tomuschat, *Between Idealism and Realism.* The Collected Courses of the Academy of European Law, Vol. 13/1 (Oxford University Press 2003) 56.

55 Christine Chinkin and Mary Kaldor, *International Law and New Wars* (OUP 2017) 565.

56 Keith Krause, ‘The Key to a Powerful Agenda, If Properly Delimited’ (2004) 35 *Security Dialogue* 367.

57 Sadako Ogata, ‘From State Security to Human Security’ (26 May 2002, Ogden Lecture, Brown University) 4 (on file with the author).

58 Commission on Human Security, *supra* note 4, 4.

59 Ibid. 4; Sabina Alkire, ‘A Conceptual Framework for Human Security’, (CRISE, Working Paper #2, 2003) <https://ora.ox.ac.uk/objects/oai:economics.ouls.ox.ac.uk:13003> 8.

60 Commission on Human Security, *supra* note 4.

61 UN General Assembly, ‘Follow-up to paragraph 143 on human security of the 2005 World Summit Outcome’, (25 October 2012) UN Doc A/RES/66/290, [3(a)] (emphasis added).

62 Martha Fineman, ‘Vulnerability, Resilience, and LGBT Youth’ (2014) 23 *Temple Political & Civil Rights Law Review* 307, 320. See also, Martha Fineman, ‘The Vulnerable Subject: Anchoring Equality in the Human Condition’ (2008) 20 *Yale Journal of Law & Feminism* 1.

63 Commission on Human Security, *supra* note 4, 11.

64 Ogata, *supra* note 57, 5.

65 Sadako Ogata, ‘The Human Security Commission’s strategy’ (2004) 16 *Peace Review* 25, 26.

66 Vanessa Friedman, ‘It’s Going to Be the Image of the Revolution’ (*New York Times*, 10 April 2019) <https://www.nytimes.com/2019/04/10/fashion/demonstration-clothing-women-sudan.html>.

67 Roland Paris, ‘Human Security: Paradigm Shift or Hot Air?’ (2001) 26(2) *International Security* 87, 88.

68 Fairlie Chappuis, ‘Human Security and Security Sector Reform’ In: Wolfgang Benedek, Matthias Kettemann and Markus Möstl (eds), *Mainstreaming Human Security in Peace Operations and Crisis Management* (Routledge 2011) 99.

69 Wolfgang Benedek, ‘Mainstreaming human security in United Nations and European Union peace and crisis management operations’ In: Wolfgang Benedek, Matthias Kettemann and Markus Möstl (eds), *Mainstreaming Human Security in Peace Operations and Crisis Management* (Routledge 2011) 13–31, 19.

70 Kjetil Mujezinović Larsen, ‘United Nations Peace Operations and International Law: What Kind of Law Promotes What Kind of Peace?’ In: Cecilia Marcela Bailliet and Kjetil Mujezinović Larsen, *Promoting Peace through International Law* (OUP 2015) 302–3.

71 See e.g. Trindade, *supra* note 10; Teitel, *supra* note 11.

72 Emmanuelle Jouannet, What Is the Use of International Law? International law as a 21st Century Guardian of Welfare’ (2006–2007) 28 *Michigan Journal of International Law* 815, 821.

73 Sari and Jachec-Neale, *supra* note 8, v.

74 Benedek, *supra* note 69, 16.

75 Oberleitner, *supra* note 54, 592–3.

76 Barbara von Tigerstrom, *Human Security and International Law* (Hart 2007) 42.

77 Gerd Oberleitner, ‘Human Security: A Challenge to International Law?’ (2005) 11(2) *Global Governance* 185, 186 (emphasis added).

78 Daft, *supra* note 52, 33.

79 Ibid. 33, 125.

80 Ibid. 86.

81 Nasu, *supra* note 16, 37.

82 UN General Assembly, ‘Follow-up to the Millennium Summit’ (4 June 2012) A/66/PV.112, 10.

83 Ibid. 2.

84 Ibid. 14.

85 Math Noortmann and Cedric Ryngaert, ‘Towards a (New) Human Security-Based Agenda for International Law and Non-State Actors’ In: Cedric Ryngaert and Math Noortmann (eds), *Human Security and International Law* (Intersensia 2014) 195–203, 198.

86 Nasu, *supra* note 16, 33.

87 Ibid. 33.

88 Daft, *supra* note 52, 82.

89 von Tigerstrom, *supra* note 76, 49.

90 Ibid. 46.

91 UN General Assembly, Informal Thematic Debate on Human Security (22 May 2008) <https://www.un.org/humansecurity/reports-resolutions/>; UN General Assembly, Informal Thematic Debate on Human Security (14 April 2011) <https://www.un.org/humansecurity/reports-resolutions/>; UN General Assembly, Informal Thematic Debate on Human Security (18 June 2014) <https://www.un.org/humansecurity/reports-resolutions/>.

92 N.b. the General Assembly can seize itself of a matter of international peace and security under certain circumstances. The ‘Uniting for Peace’ Resolution (Resolution 377A (3 November 1950)) states that in a situation where the Security Council is unable to exercise its primary responsibility for the maintenance of international peace and security then the General Assembly may immediately consider the matter at hand and make recommendations to maintain or restore international peace and security. This was used in 1956 to create UNEF I but missions are, under most circumstances, created by the UN Security Council under the auspices of Chapter VI and VII of the UN Charter.

93 Daft, *supra* note 52, 71.

94 Trina Ng, ‘Safeguarding Peace and Security in our Warming World: A Role for the Security Council’ (2010) 15 *Journal of Conflict and Security Law* 275, 280.

95 Barry Buzan, ‘Human Security in International Perspective’ In: Mely Caballero-Anthony, Jawhar Hassan (eds), *The Asia Pacific in the New Millennium: Political and Security Challenges* (Institute of Strategic and International Studies 2001) 583–96.

96 Shahrbanou Tadjbakhsh, ‘Human Security: Concepts and Implications with an Application to Post-Intervention Challenges in Afghanistan’ (Les Études du CERI No. 117–118, Sciences Po, 2005) 9. See also, Shahrbanou Tadjbakhsh, Anuradha Chenoy, *Human Security: concepts and implications* (Routledge 2007).

97 Hideaki Shinoda, ‘The Concept of Human Security: Historical and Theoretical Implications’ (IPSHU English Research Report Series No. 19, Conflict and Human Security: A Search for New Approaches of Peace-building, 2004) 13.

98 Tadjbakhsh, *supra* note 96, 24.

99 Ibid. 24.

100 Ibid. 25.

101 Ibid. 25.

102 Brendan Howe, Boris Kondock and Otto Spijkers, ‘Normative and Legal Challenges to UN Peacekeeping Operations’ (2015) 19 *Journal of International Peacekeeping* 1–31, 30.

103 UN Security Council, ‘Report of the Secretary-General on the situation in Mali’ (10 June 2013) UN Doc S/2013/338 [40, 82]; UN Security Council, ‘Report of the Secretary-General on the Situation in Mali’ (26 December 2017) UN Doc S/2017/1105 [49]; UN Security Council, ‘Report of the Secretary-General on the Situation in Mali’ (28 September 2017) UN Doc S/2017/811 [47].

104 UN Security Council, Resolution 2227 (29 June 2015) UN Doc S/RES/2227 [14(e)]; UN Security Council, Resolution 2295 (29 June 2016) UN Doc S/RES/2295 [19(f)]; UN Security Council, Resolution 2364 (29 June 2017) UN Doc S/RES/2364 [20(f)].

105 UN Security Council, ‘Letter dated 19 December 2014 from the Secretary-General addressed to the President of the Security Council’ (22 December 2014) S/2014/928 [63].

106 See e.g. UN Security Council, ‘Report of the Secretary-General on the situation in the Central African Republic’ (1 April 2015) S/2015/227 [47]; UN Security Council, ‘Report of the Secretary-General on the situation in Mali’ (9 June 2014) UN Doc S/2014/403 [28]; S/2017/811, *supra* note 103, [59].

107 UN Security Council, ‘Report of the Secretary-General on the situation in Mali’ (28 November 2012) UN Doc S/2012/894 [12].

108 UN Security Council, ‘Report of the Secretary-General on the situation in Mali’ (22 September 2015) UN Doc S/2015/732 [33]; UN Security Council, ‘Situation in the Central African Republic’ (18 June 2018) S/2018/611 [40].

109 UN Security Council, ‘Situation in the Central African Republic’ (1 April 2016) S/2016/305 [26].

110 UN Security Council, ‘Report of the Secretary-General on the Situation in Mali’ (6 June 2017) UN Doc S/2017/478 [64].

111 See e.g. UN Security Council, Resolution 2100 (25 April 2013) UN Doc S/RES/2100 [16(c)(ii)].

112 Ibid. [2]; UN Security Council, Resolution 2164 (25 June 2014) UN Doc S/RES/2164 [16].

113 UN Security Council, ‘Report of the Secretary-General on the Central African Republic submitted pursuant to paragraph 48 of Security Council resolution 2127 (2013)’ (3 March 2014) S/2014/142 [14].

114 UN Security Council, Resolution 2217 (28 April 2015) S/RES/2217, 4.

115 UN Security Council, ‘Letter dated 29 January 2015 from the Secretary-General addressed to the President of the Security Council’ (29 January 2015) S/2015/85, 2; UN Security Council, ‘Report of the Secretary-General on the situation in the Central African Republic’ (29 July 2015) S/2015/576 [20].

116 UN Security Council, ‘Report of the Secretary-General on the situation in Mali’ (28 March 2014) UN Doc S/2014/229 [46]; Helené Lackenbauer, Magdalena Tham Lindell and Gabriella Ingerstad, ‘“If our men won’t fight, we will” A Gendered Analysis of the Armed Conflict in Northern Mali’ (Swedish Defence Research Agency, November 2015, FOI-R-4121-SE) 43.

117 S/RES/2100, *supra* note 111, [16(c)(i)]; UN Security Council, Resolution 2149 (10 April 2014) S/RES/2149 [30(a)].

118 On the topic of robust force see e.g. Alexander Gilder, ‘The Effect of ‘Stabilization’ in the Mandates and Practice of UN Peace Operations’ (2019) *Netherlands International Law Review* 66(1) 47–73.

119 UN Security Council, ‘Report of the Secretary-General on the Situation in Mali’ (1 October 2013) UN Doc S/2013/582 [44]; UN Security Council, ‘Letter dated 29 July 2016 from the Permanent Representatives of Spain and the United Kingdom of Great Britain and Northern Ireland to the United Nations addressed to the Secretary-General’ (8 August 2016) UN Doc S/2016/682, 3; UN Security Council, ‘Report of the Secretary-General on the situation in Mali’ (30 December 2016) UN Doc S/2016/1137 [37]; UN Security Council, ‘Report of the Secretary-General on the situation in the Central African Republic’ (1 August 2014) S/2014/562 [48]; UN Security Council, ‘Report of the Secretary-General on the situation in the Central African Republic’ (28 November 2014) S/2014/857 [45]; S/2015/227, *supra* note 106, [45].

120 S/RES/2149, *supra* note 117, [30(b)(v)]; S/RES/2100, *supra* note 111, [16(b)(iii), 25]; S/RES/2295, *supra* note 104, [26].

121 UN Security Council, Resolution 2056 (5 July 2012) UN Doc S/RES/2056 [26].

122 UN Security Council, ‘Report of the Secretary-General on the situation in the Central African Republic’ (1 February 2017) S/2017/94 [21].

123 Commission on Human Security, *supra* note 4, 11.

124 UN Security Council, ‘Report of the Secretary-General on the Central African Republic’ (2 June 2017) S/2017/473 [21].

125 Sara E Davies and Simon Rushton, ‘Public health emergencies: a new peacekeeping mission? Insights from UNMIL’s role in the Liberia Ebola outbreak’ (2016) 37(3) *Third World Quarterly* 419–435.

126 UN Security Council, Resolution 2177 (18 September 2014) UN Doc S/RES/2177.

